# LRP-2 controls the localization of *C. elegans* SYS-1/beta-catenin

**DOI:** 10.17912/micropub.biology.000151

**Published:** 2019-08-27

**Authors:** Paul J Minor, Paul W Sternberg

**Affiliations:** 1 Division of Biology and Biological Engineering, Caltech, Pasadena, CA 91125; 2 Department of Biology, Hopkins Marine Station of Stanford University, Pacific Grove, CA 93950

**Figure 1. LRP-2 controls the asymmetric localization of SYS-1:**
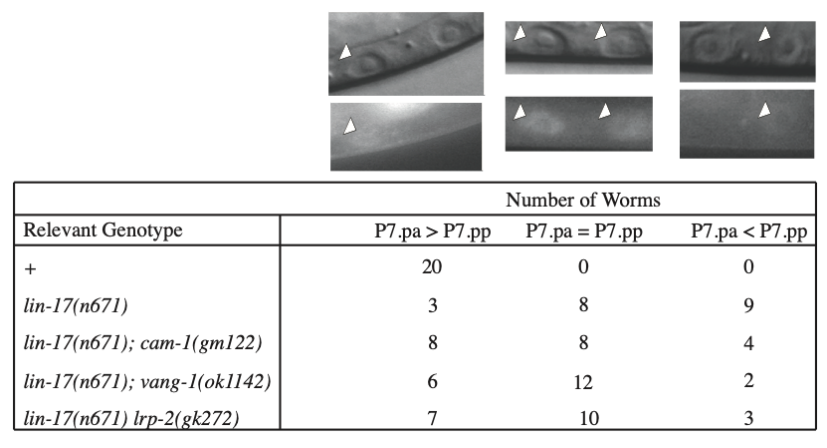
The localization pattern of VNS::SYS-1 in P7.p daughter cells. The resulting pattern was classified by eye into three categories: SYS-1 enriched in the anterior daughter (P7.pa > P7.pp), SYS-1 present at similar levels in both daughters (P7.pa = P7.pp), and SYS-1 enriched in the anterior daughter (P7.pa < P7.pp). A representative image of each scenario is shown.

## Description

The polarity of the *C. elegans* P7.p cell divisions is controlled by the Wnt/β-catenin asymmetry pathway (Green *et al*., 2008; Minor *et al*., 2013). This pathway includes the β-catenin-like proteins SYS-1 and WRM-1, POP-1/TCF, and the Nemo-like-kinase, LIT-1 (reviewed by Mizumoto and Sawa, 2007). The Wnt/β-catenin asymmetry pathway ensures different ratios of SYS-1 to POP-1, controlling the differential transcription of Wnt target genes between daughters of an asymmetric cell division. Because our genetic data indicate an antagonism between LRP-2 and LIN-17 similar to that between CAM-1 and VANG-1 and LIN-17 (Minor and Sternberg, 2019), we wanted to determine if LRP-2 can control the asymmetric localization of SYS-1 between the daughter cells of P7.p during anaphase of the first cell division. The initial establishment of vulval polarity can be observed through the localization of VENUS::SYS-1 (VNS::SYS-1), localized in a high (P7.pa)/low (P7.pp) pattern in the wild-type worm, reciprocal to the localization of POP-1/TCF (Phillips *et al*., 2007; Green *et al*., 2008).

It was previously reported (Green *et al*. 2008) that VNS::SYS-1 asymmetry in P7.p daughter cells is often lost in *lin-17(n671)* and *lin-18(e620)* mutants. These mutants display two aberrant patterns of VNS::SYS-1 localization as well as the wild-type pattern, though less frequently. The two deviant localization patterns include one in which both P7.pa and P7.pp express equal amounts of VNS::SYS-1 and a reversed VNS::SYS-1 pattern in which P7.pp is enriched with VNS::SYS-1. By observing VNS::SYS-1 localization in a *lin-17(n671); lrp-2(gk272)* background we see that the aberrant localization of SYS-1 is suppressed to a similar degree to that of *lin-17(n671); cam-1(gm122)* and *lin-17(n671); vang-1(ok1142)*. This observation confirms LRP-2 controls vulval cell polarity by antagonizing LIN-17 in a similar fashion to CAM-1 and VANG-1, and that the effect of LRP-2 is at the level of P7.p rather than its progeny.

## Reagents

**Strains:**

N2

**MT1306**: *lin-17(n671)* (Ferguson and Horvitz, 1985)

**MT1488**: *lin-17(n671); unc-13(e1091)*

**PS5840**: *lin-17(n671); cam-1(gm122); qIs95[pSYS-1::VENUS::SYS-1]* (Green *et al*., 2008)

**PS5787**: *lin17(n671); vang-1(ok1142); qIs95[pSYS-1::VENUS::SYS-1]* (Green *et al*., 2008)

The *lin17(n671); lrp-2(gk272)* double mutant was constructed by crossing **VC543** *lrp-2(gk272)* males with strain **MT1488**: *lin-17(n671); unc-13(e1091)* hermaphrodites.

**JK4062**: *lin-17(n671); qIs95[pSYS-1::VENUS::SYS-1]*

The *lin17(n671); lrp-2(gk272); qIs95[pSYS-1::VENUS::SYS-1]* line was created by crossing **VC543** *lrp-2(gk272)* males with **JK4062**: *lin-17(n671); qIs95[pSYS-1::VENUS::SYS-1]* hermaphrodites
